# Supplementary material for: Delineating the Dynamic Transcriptome Response of mRNA and microRNA during Zebrafish Heart Regeneration
Source: Biomolecules. 2018 Dec 28;9(1):11. doi: 10.3390/biom9010011 (PMC6359357; doi:10.3390/biom9010011)
Supplement: Supplementary file 1 [file biomolecules-09-00011-s001.zip › Figure S3.pdf]

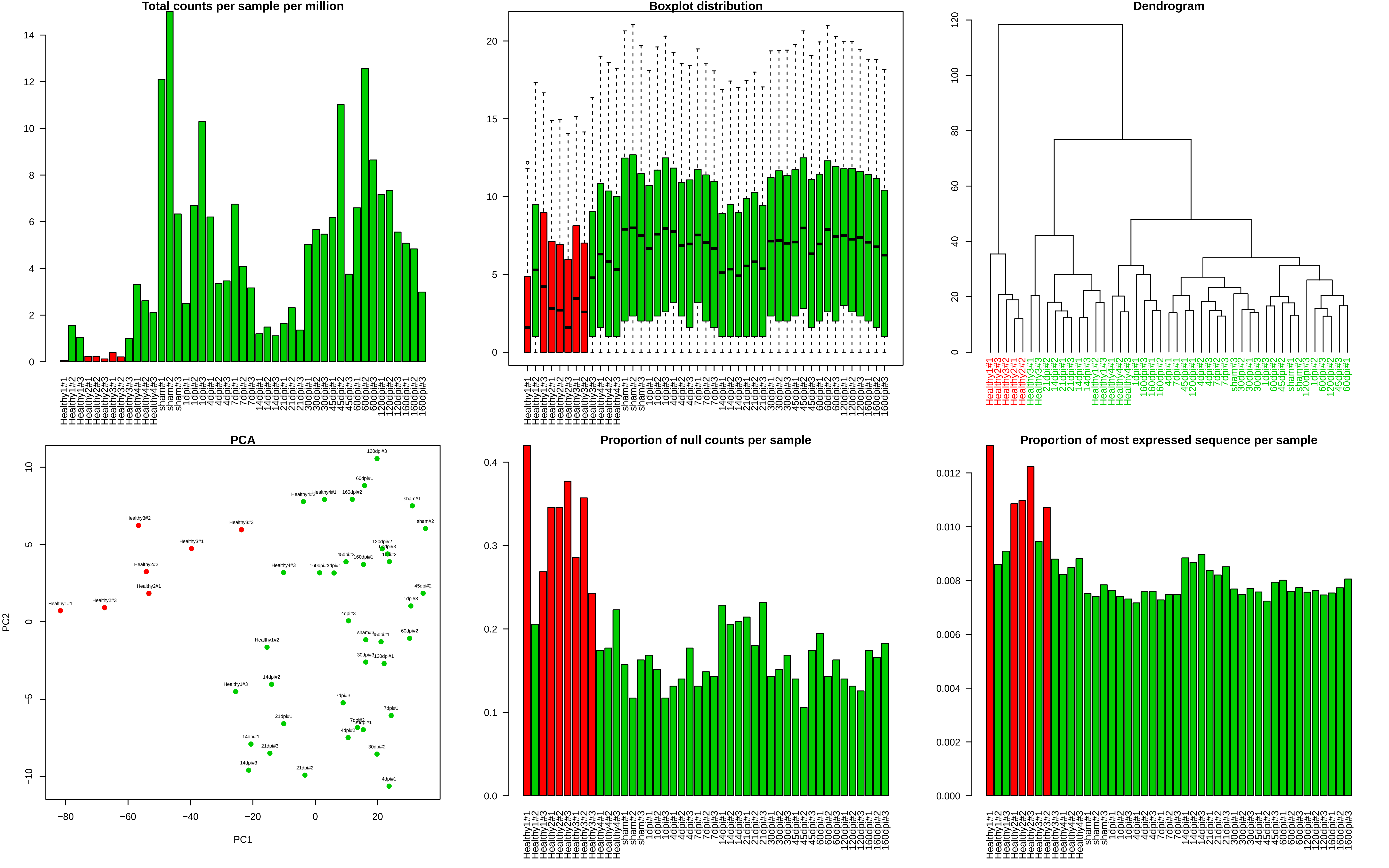
Total counts per sample per million
Boxplot distribution
Dendrogram
PCA
PC1
PC2
Proportion of null counts per sample
Proportion of most expressed sequence per sample
