# Supplementary figures and images for: Delineating the Dynamic Transcriptome Response of mRNA and microRNA during Zebrafish Heart Regeneration
Source: Biomolecules. 2018 Dec 28;9(1):11. doi: 10.3390/biom9010011 (PMC6359357; doi:10.3390/biom9010011)

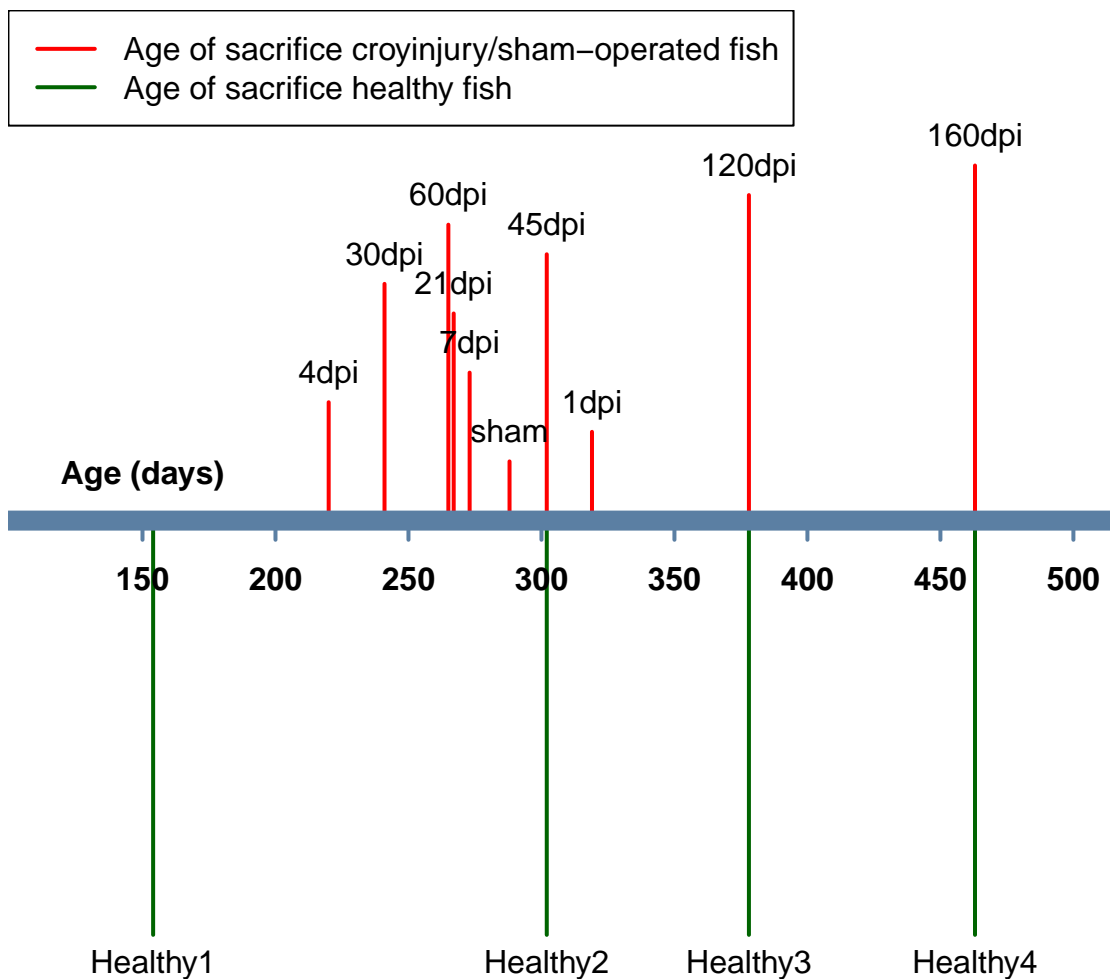

Supplement: Supplementary file 1 [file biomolecules-09-00011-s001.zip › Figure S1.pdf]

## miRNA read counts in H9c2 cell line

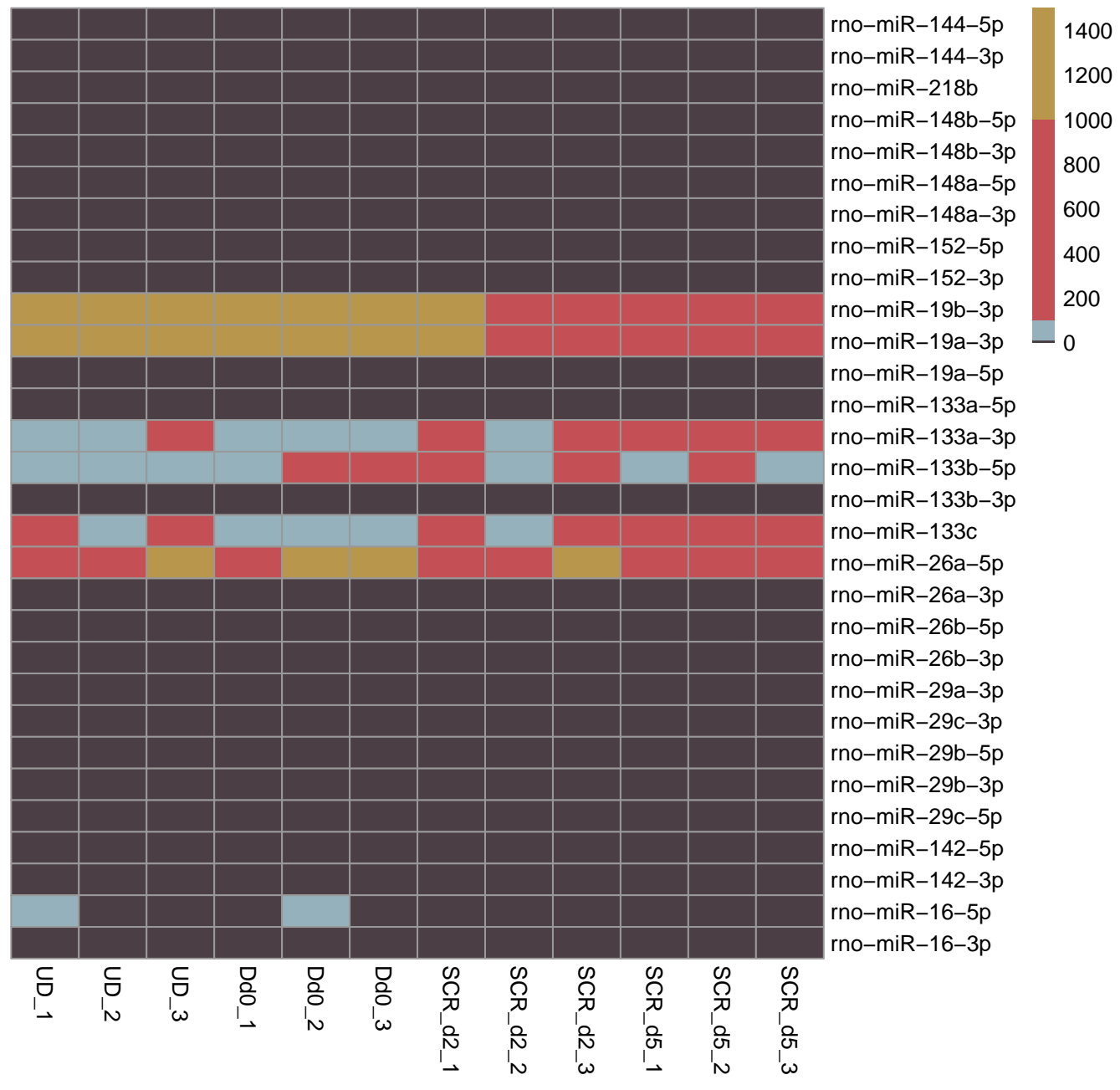

Supplement: Supplementary file 1 [file biomolecules-09-00011-s001.zip › Figure S6.pdf]

Data before batch correction

mRNA

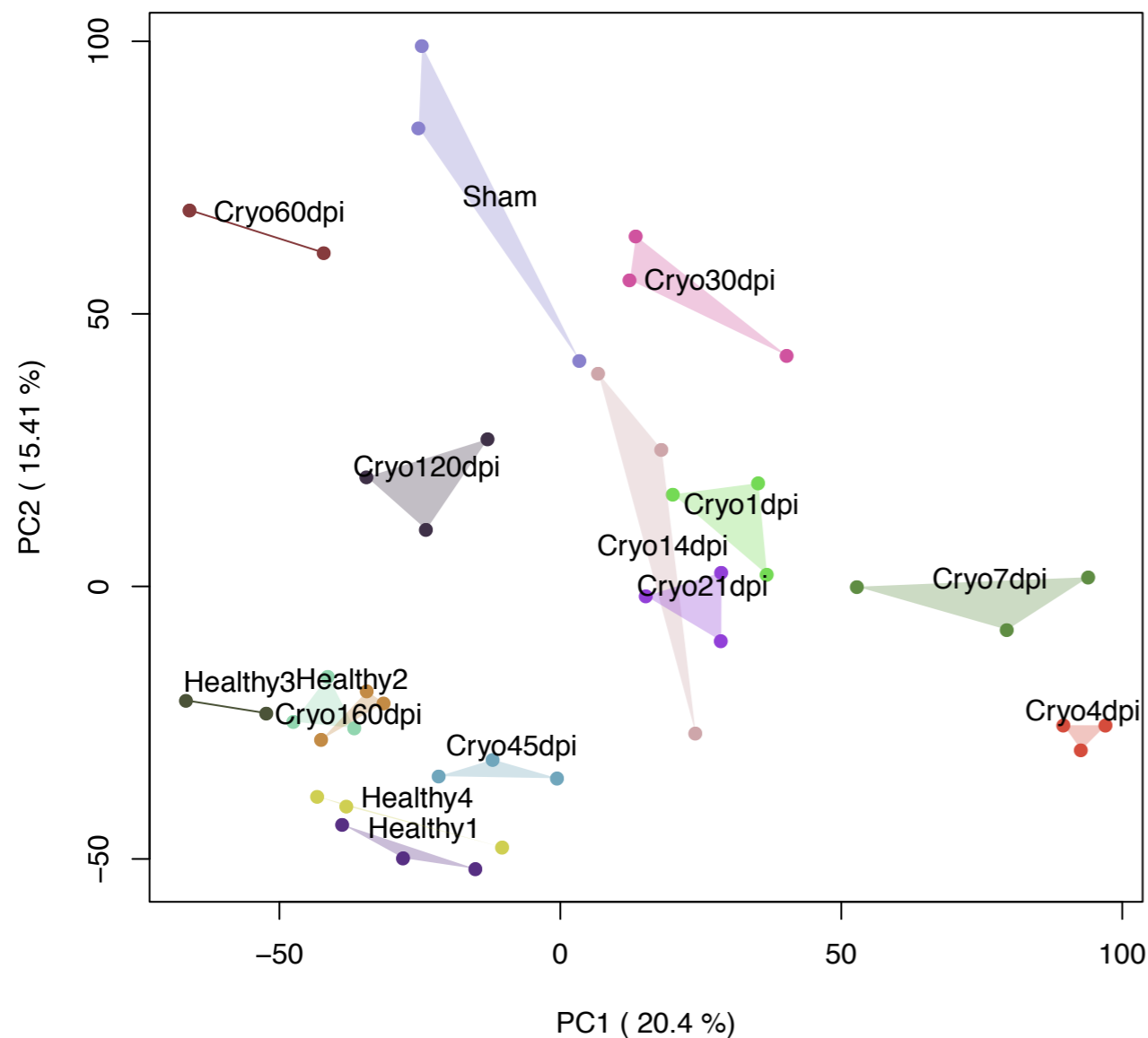

SVA

Data after batch correction

PC2 ( 15.5 %)

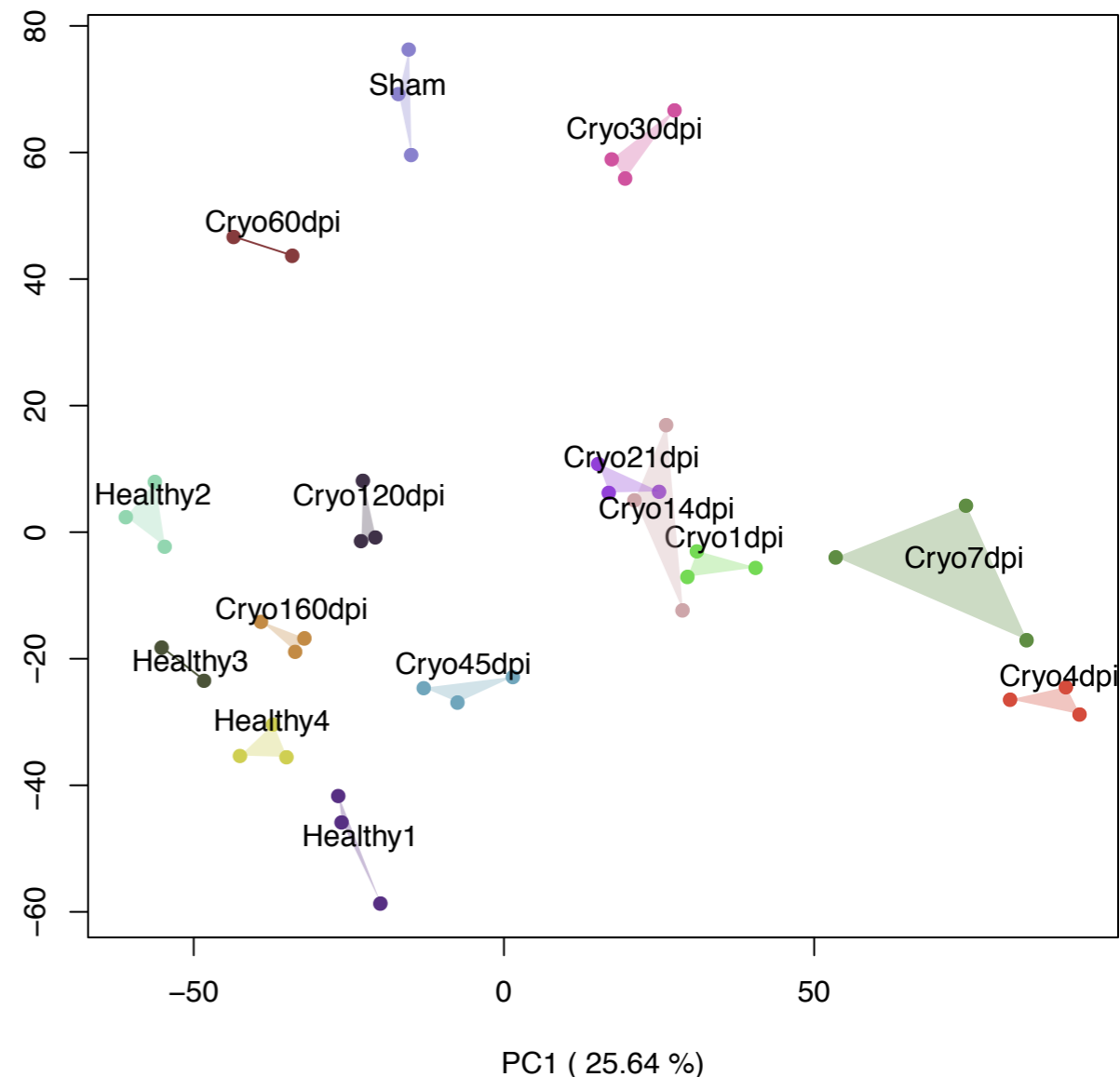

miRNA

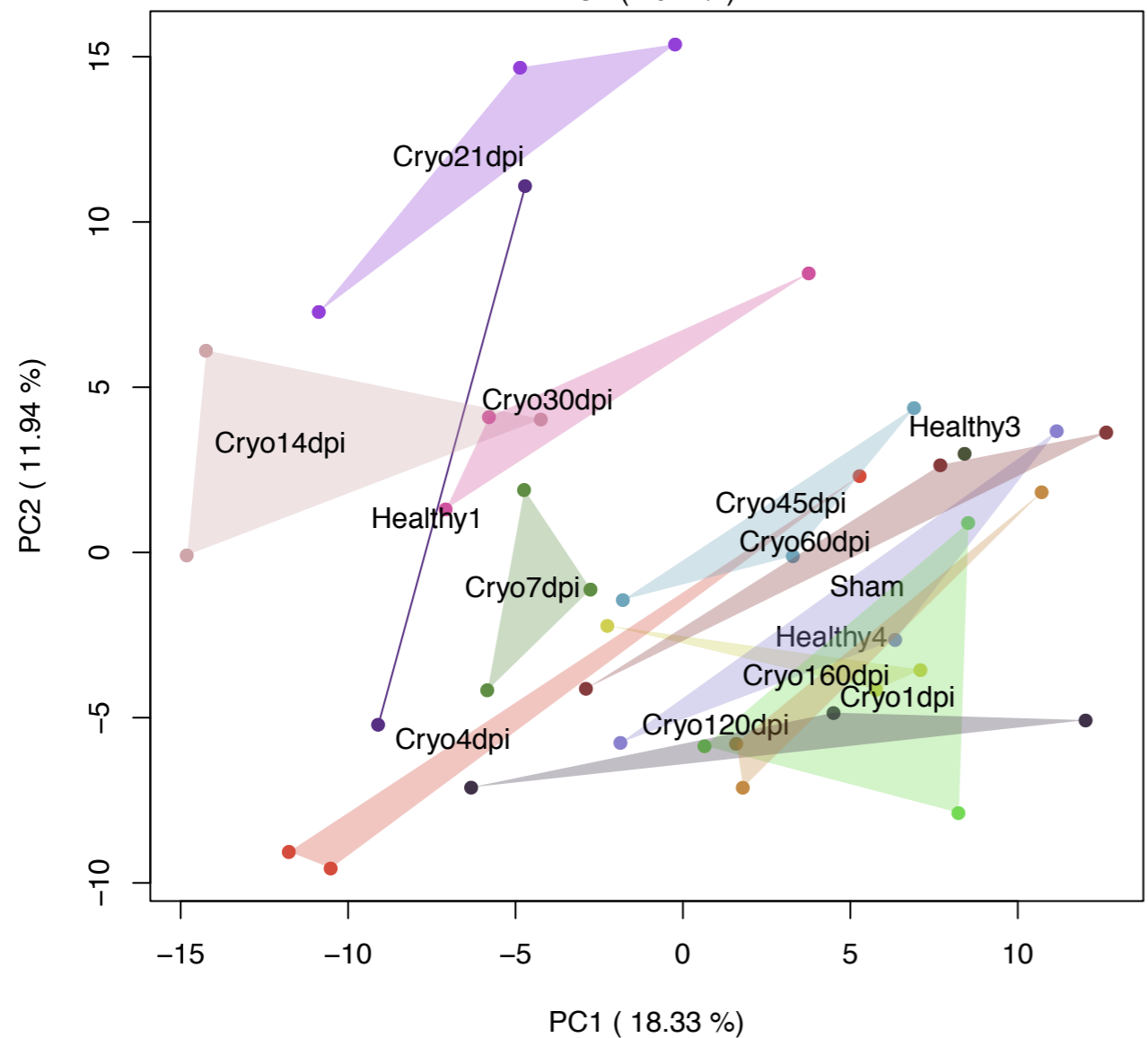

SVA

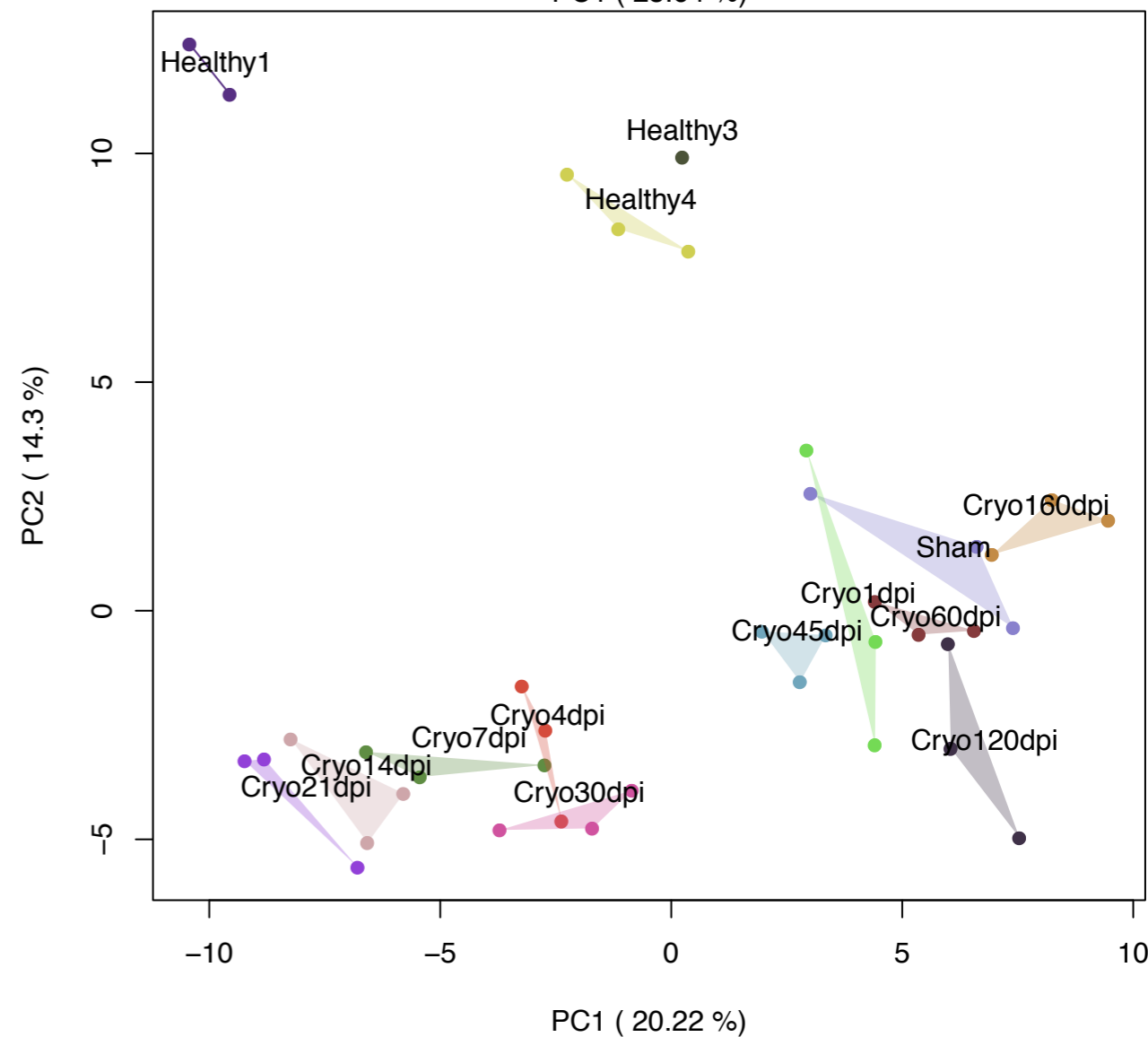

Supplement: Supplementary file 1 [file biomolecules-09-00011-s001.zip › Figure S4.pdf]

**miR-144**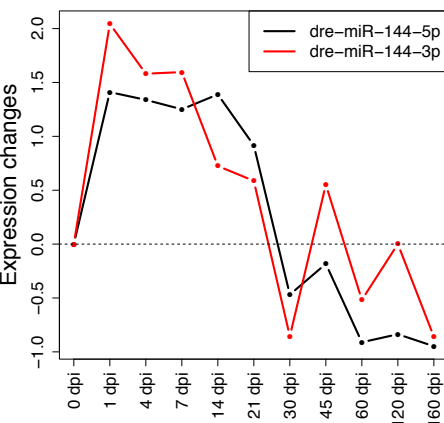**miR-26ab**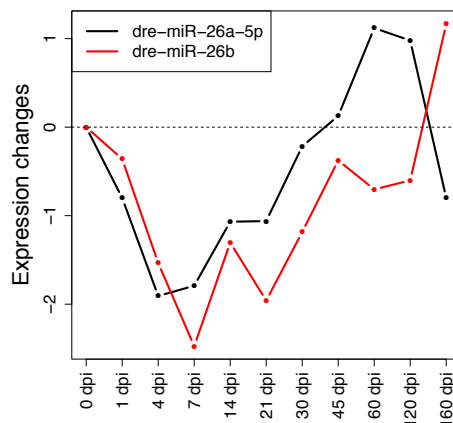**miR-218ab**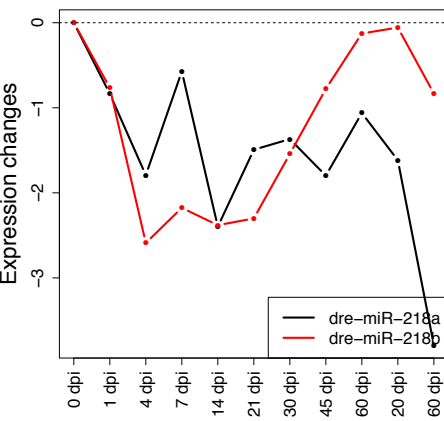**miR-29b**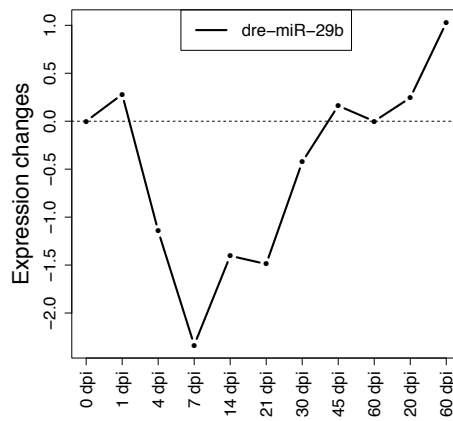**miR-148/miR-152**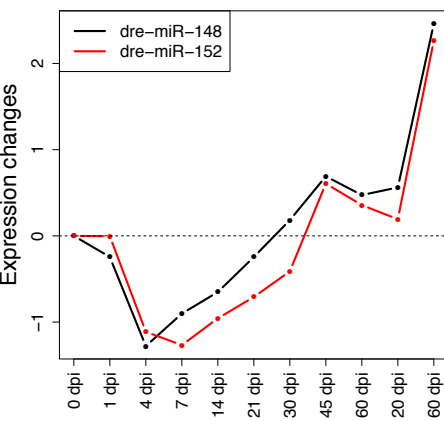**miR-101ab**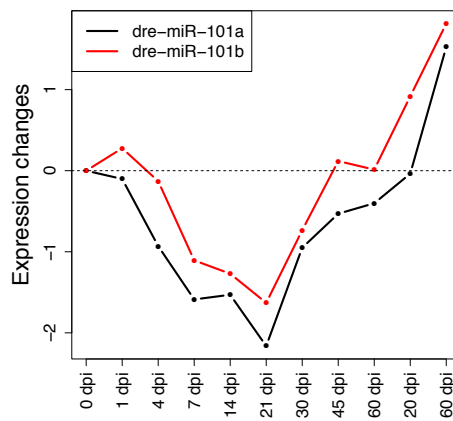**miR-19abcd**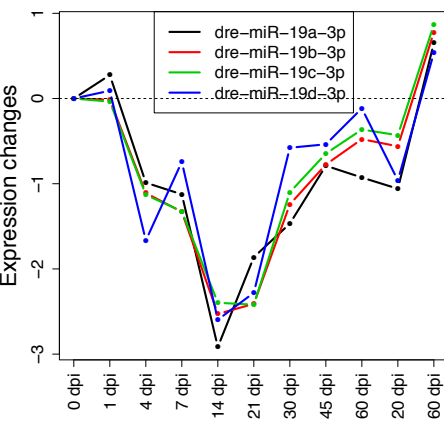**miR-142a**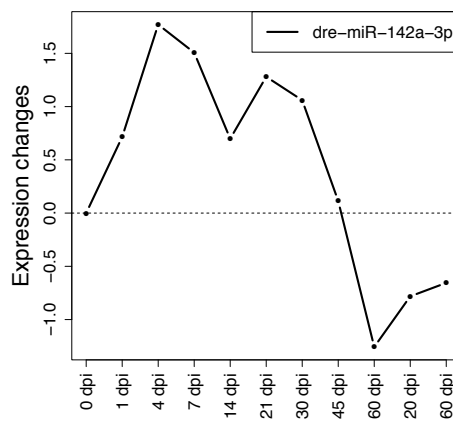**miR-133abc**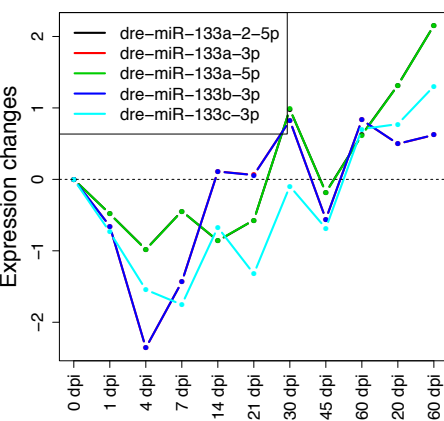**miR-16c**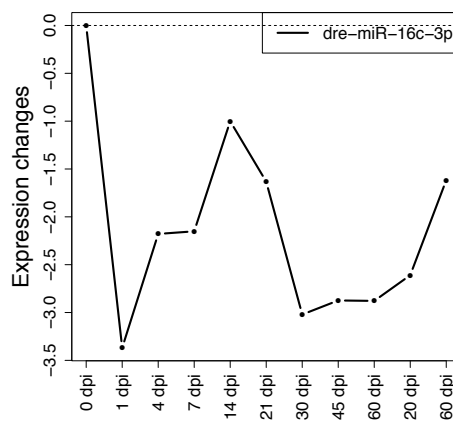

Supplement: Supplementary file 1 [file biomolecules-09-00011-s001.zip › Figure S5.pdf]
